# Predicting erythropoietin resistance in hemodialysis patients with type 2 diabetes

**DOI:** 10.1186/1471-2369-14-67

**Published:** 2013-03-22

**Authors:** Andreas Schneider, Markus P Schneider, Hubert Scharnagl, Alan G Jardine, Christoph Wanner, Christiane Drechsler

**Affiliations:** 1 Department of Medicine, Division of Nephrology, University Hospital Wuerzburg, Wuerzburg, Germany; 2 Renal Research Group, British Heart Foundation (BHF) Cardiovascular Research Centre, Institute of Cardiovascular and Medical Sciences, University of Glasgow, Glasgow, UK; 3 Department of Nephrology and Hypertension, University Hospital Erlangen, Erlangen, Germany; 4 Clinical Institute of Medical and Chemical Laboratory Diagnostics, Medical University Graz, Graz, Austria

## Abstract

**Background:**

Resistance to ESAs (erythropoietin stimulating agents) is highly prevalent in hemodialysis patients with diabetes and associated with an increased mortality. The aim of this study was to identify predictors for ESA resistance and to develop a prediction model for the risk stratification in these patients.

**Methods:**

A post-hoc analysis was conducted of the 4D study, including 1015 patients with type 2 diabetes undergoing hemodialysis. Determinants of ESA resistance were identified by univariate logistic regression analyses. Subsequently, multivariate models were performed with stepwise inclusion of significant predictors from clinical parameters, routine laboratory and specific biomarkers.

**Results:**

In the model restricted to clinical parameters, male sex, shorter dialysis vintage, lower BMI, history of CHF, use of ACE-inhibitors and a higher heart rate were identified as independent predictors of ESA resistance. In regard to routine laboratory markers, lower albumin, lower iron saturation, higher creatinine and higher potassium levels were independently associated with ESA resistance. With respect to specific biomarkers, higher ADMA and CRP levels as well as lower Osteocalcin levels were predictors of ESA resistance.

**Conclusions:**

Easily obtainable clinical parameters and routine laboratory parameters can predict ESA resistance in diabetic hemodialysis patients with good discrimination. Specific biomarkers did not meaningfully further improve the risk prediction of ESA resistance. Routinely assessed data can be used in clinical practice to stratify patients according to the risk of ESA resistance, which may help to assign appropriate treatment strategies.

**Clinical trial registration:**

The study was registered at the German medical authority (BfArM; registration number 401 3206). The sponsor protocol ID and clinical trial unique identified number was CT-981-423-239. The results of the study are published and available at http://www.ncbi.nlm.nih.gov/pubmed/16034009.

## Background

Despite advances in renal replacement therapy, mortality of hemodialysis (HD) patients is still excessive [1]. Diabetes mellitus is the leading cause of kidney disease. Almost half of US dialysis patients developed end-stage renal disease due to diabetes mellitus. Compared to non-diabetic dialysis patients, diabetic dialysis patients show strikingly higher mortality rates which is reflected by a five year survival of only 35% [2].

Anemia is one of the major problems contributing to the high comorbidity and poor outcome of diabetic dialysis patients. Anemia treatment in chronic kidney disease (CKD) patients has changed dramatically since the implementation of Erythropoietin Stimulating Agents (ESAs) into clinical practice in 1989. This has reduced the need for blood transfusions, improving quality of life for the patients [3]. ESA resistance has been defined by the European Renal Association-European Dialysis and Transplant Association (ERA-EDTA) as being present when patients do not achieve the recommended hemoglobin (Hb) target level (11–12 g/dl), despite a treatment with ESAs over several months [4]. According to this arbitrary definition, more than 90–95% of HD patients treated with an ESA respond to the therapy with a sufficient rise in the hemoglobin value [5], whereas 5–10% do not adequately respond to the therapy. Of note, resistance to ESAs has consistently been shown to be associated with an increased risk of death and cardiovascular events in CKD patients [6-8]. In the recent TREAT trial, diabetic patients with CKD were at highest risk of mortality when they had a poor response to the initial two doses of darbepoietin alfa [6]. Besides, ESA therapy is expensive and leads to enormous costs for the Health Care Systems [9]. Therefore, strategies to reduce ESA resistance and to avoid unnecessary ESA usage are required. In clinical practice, tools to identify patients who most likely will benefit from ESA therapy would be highly useful.

Several factors have been described to promote ESA resistance in HD patients [7,10-13]. By now, inflammation, malnutrition, secondary hyperparathyroidism (sHPT), lower hemoglobin A1C (HbA1c), deplete iron stores and vitamin D deficiency have been found to be associated with ESA resistance. However, the combination of known risk factors enabling to stratify patients into responders and non-responders has not been investigated so far. Hence, we developed a model predicting ESA resistance in HD patients utilizing data of the prospective German Diabetes and Dialysis Study (4D - Die Deutsche Diabetes Dialyse-Studie) [14].

## Methods

### Design of the 4D study

The 4D study design, main outcome findings, and baseline data have been described previously [14]. In short, the 4D study was a prospective randomized controlled trial recruiting 1255 hemodialysis patients with type 2 diabetes mellitus, aged 18–80 years, from 178 German dialysis centres. Eligible patients were randomly assigned to receive either atorvastatin 20 mg daily or matching placebo. The mean length of follow-up was 4.0 years. The primary end point of the 4D study was defined as a composite of death from cardiac causes, stroke and myocardial infarction. All events were reviewed and adjudicated by a critical end point committee blinded to treatment allocation [14].

The study adhered to the International Conference on Harmonisation guidelines for Good Clinical Practice and was conducted in accordance with the Declaration of Helsinki. The protocol was approved by the ethics committee at the University of Würzburg.

All participants provided written informed consent.

### Data collection

Information on demographic characteristics such as age and smoking status were obtained through patient interviews. Comorbidities including the presence of coronary artery disease (CAD) and congestive heart failure (CHF), as well as the duration of diabetes mellitus and dialysis treatment, were reported by the patients’ nephrologists. Blood pressure was measured in a sitting position. Body mass index (BMI) was calculated as weight (kilogram) divided by height (metre squared).

### ESA resistance

In the present study we calculated the ESA Resistance Index (ERI), defined as the weekly weight-adjusted ESA dose (U/kg/week) divided by hemoglobin level (g/dl). By means of the ERI values (baseline data), patients were divided into quartiles. The cut-off values were Quartile 1 < 4.19, Quartile 2 (4.20 – 6.64), Quartile 3 (6.65 – 10.11) and Quartile 4 > 10.11, respectively. Patients in the upper quartile were defined as ESA resistant. The ERI index was calculated population-based in a cross-sectional fashion at baseline.

### Statistical analyses

Continuous variables were expressed as mean with standard deviation (SD), and categorical variables were expressed as percentages. We investigated potential predictors of ESA resistance. Firstly, based on previous knowledge from the literature and clinical relevance, we evaluated demographic and clinical characteristics (age, sex, body mass index, smoking status, systolic and diastolic blood pressure, dialysis vintage, duration of diabetes mellitus), comorbidities (CAD, CHF, peripheral vascular disease), and the use of concomitant medication (ACE-inhibitors, beta-blockers, diuretics).

Secondly, we investigated potential predictors from baseline routine laboratory assessments including hemoglobin, albumin, calcium, phosphate, potassium, urea, HbA1c, alkaline phosphatase, iron and lipid status. Finally, specific non-routinely used biomarkers were additionally evaluated including N-terminal-pro-B-type-natriuretic peptide (NT-pro-BNP), C-reactive protein, parathyroid hormone (PTH), adiponectin, vitamin D (25(OH)D), troponin T, osteocalcin, ADMA, osteoprotegerin and fetuin.

Variables were log transformed if necessary. We performed univariate logistic regression analyses to assess the association of clinical and laboratory markers with the binary outcome of ESA resistance (yes/no). Odds ratios (OR) and 95% confidence intervals were calculated.

Furthermore, we performed multivariate logistic regression analyses to construct a prediction model for ESA resistance. Predictors with a p-value < 0.1 in univariate analyses were selected for inclusion in the multivariate model. Backward selection procedures were applied in the multivariate analyses. The construction of the prediction model was performed in several steps. First, we established a prediction model based on the clinical parameters described above (model 1) and investigated whether the addition of routine laboratory markers measured at baseline (model 2) and specific biomarkers (model 3) further improved risk stratification for ESA resistance. Prognostic indices of the models were assessed and. analyses were performed using SPSS version 19.0.

## Results

### Patient characteristics at baseline

Altogether, 1255 patients were included into the 4D study, of which 1015 had a measurement of the ESA-Index at baseline. Table 1 shows the patients baseline characteristics.

**Table 1:** Baseline patient characteristics, study population n = 1015

**Characteristic**	**n**	**All**	**Non epo-resistant**	**Epo-resistant**
Age *years*	1015	66 (8)	65 (8)	67 (8)
Gender *% men*	1015	53.9	55	41
Systolic BP *mmHg*	1015	145 (22)	146 (23)	147 (23)
Diastolic BP *mmHg*	1015	76 (11)	76 (11)	75 (11)
BMI *kg/m*^*2*^	1015	27.5 (4.8)	27.6 (4.8)	26.9 (5.0)
Duration of diabetes *years*	953	18.1 (8.8)	17.6 (8.7)	18.5 (8.7)
Time on dialysis *months*	1015	8.2 (6.9)	8.6 (7.0)	7.0 (6.5)
History of				
CAD %	1015	28,50	28,50	28,50
CHF *%*	1015	35.4	32.2	41.9
PVD *%*	1015	44.6	43.8	46.6
Smoker or ex-smoker *%*	1015	40.4	40.8	34.4
use of ACE-inhibitors %	1015	48	48	54
heart rate bpm	988	79 (15)	78 (15)	81 (17)
Laboratory parameters	253	25	0	100
ERI-Index > 10.1 (%)*				
LDL cholesterol *mg/dl*	1015	126 (30)	126 (31)	121 (27)
AP U/I	1015		123 (63)	
HDL cholesterol *mg/dl*	1015	36 (13)	36 (13)	37 (15)
Triglycerides *mg/dl*	1015	264 (167)	269 (173)	260 (172)
Hemoglobin *g/dl*	1015	10.9 (1.3)	11.0 (1.2)	10.3 (1.5)
Albumin *g/dl*	1015	3.8 (0.3)	3.8 (0.3)	3.7 (0.3)
C-reactive protein *mg/l*	1009	5.8 (2.3-12.4)	9.9 (15.8)	14.7 (20.8)
Calcium *mmol/l*	1015	2.3 (0.2)	2.3 (0.2)	2.3 (0.2)
Phosphate *mg/dl*	1015	6.1 (1.6)	6.1 (1.6)	6.2 (1.6)
HbA1c %	1015	6.7 (1.3)	6.7 (1.2)	6.6 (1.3)
urea mg/dl	1015	143 (36)	144 (36)	137 (35)
potassium mmol/l	1015	5.2 (0.9)	5.2 (0.8)	5.4 (1.0)
ferritin μg/L	1015	487 (426)	520 (436)	442 (405)
iron saturation %	1015	22.2 (11.3)	23.3 (11.5)	17.9 (8.7)
NT-pro-BNP *pg/ml*	1009	3361 (1433–9271)	7885 (14153)	9609 (14105)
Troponin T *ng/ml*	1009	0.09 (0.13)	0.08 (0.11)	0.11 (1.17)
ADMA umol/l	1007	0.87 (0.16)	0.86 (0.15)	0.90 (0.16)
PTH pg/mL	1008	102 (119)	101 (117)	91 (127)
25(OH)Vitamin D ng/mL	1015	17.9 (9.8)	18.2 (10.1)	16.8 (8.8)
Osteocalcin ug/l	1008	107 (102)	109 (100)	101 (100)

Values are presented as mean ± SD or percentages.

Abbreviations: CAD = coronary artery disease; CHF = congestive heart failure, PVD = peripheral vascular disease.

BMI = body-mass index, LDL = low-density lipoprotein, HDL = high-density lipoprotein.

HbA1c = glycosylated hamoglobin, NT-pro-BNP = N-terminal pro b-type natriuretic peptide.

* ESA Resistance Index.

Cut-off values: Q1 < 4.19; Q2 (4,20-6.64); Q3 (6.65 – 10.11); Q4 > 10.11.

Patients in the upper ERI quartile were defined as ESA resistant.

Patients had a mean age of 66 years and 54% were men. About one third of the patients had CAD or CHF. More than half of the patients were non-smokers. All patients had diabetes mellitus and underwent HD treatment; the mean duration of diabetes was 18 years and dialysis vintage 8 months. Patients with ESA resistance had a significantly higher incidence of CVE by 33% as compared to non-resistant patients during the median follow-up of 4 years (HR 1.33, 95% CI 1.06-1.67, p = 0.013). Mortality was even significantly increased by 44% and highest with a 77% increase in the first year of follow-up (HR 1.77, 95% CI 1.23-2.54, p = 0.002).

### Predictors of ESA resistance from clinical parameters

We first analysed the role of clinical parameters for ESA resistance. This was done as information on patients demographic characteristics is easy accessible in the daily clinic routine and free of additional costs. In the study population (n = 1015), univariate analyses revealed that older age, male sex, previous or current smoking and a shorter dialysis vintage were associated with ESA resistance (Table 2). Furthermore, a lower BMI, higher heart rate, the presence of CHF and the use of ACE-inhibitors were associated with ESA resistance (all p-value <0.1). These parameters were selected for inclusion in the multivariate model. After the application of backward selection procedures, male sex, BMI, dialysis vintage, heart rate, CHF and use of ACE inhibitors remained in the final model of clinical parameters (model 1) to predict ESA resistance (Table 3). On the other hand, no associations were found between ESA resistance and blood pressure, hypertension (yes/no), PVD, arrhythmia, ultrafiltration volume, polyneuropathy, retinopathy or the duration of hemodialysis treatment per week.

**Table 2:** Determinants for ESA resistance (Q4 ERI > 10.1): univariate analyses

**Characteristic**	**OR**	**95**% **CI (lower and upper limit)**		**P-value**
Age *years*	1,02	1,00	1,04	0,05
Male sex	1,76	1,32	2,35	<0,001
Time on HD *months*	0,96	0,94	0,99	0,002
Smoker or ex-smoker	0,76	0,57	1,02	0,07
BMI kg/m^2^	0,97	0,94	1,00	0,06
Heart Rate *bpm*	1,01	1,00	1,02	0,02
History of CHF %	1,52	1,14	2,04	0,01
Use of ACE-Inhibitors %	1,27	0,96	1,69	0,09
Laboratory values				
Iron saturation *%*	0,94	0,93	0,96	<0,001
Ferritin *ug/l*	1,00	0,99	1,00	0,01
Albumin *g/dl*	0,22	0,14	0,36	<0,001
Urea *mg/dl*	0,99	0,99	1,00	0,01
Potassium *mmol/l*	1,24	1,05	1,46	0,01
Glucose *mg/dl*	1,00	1,00	1,01	0,03
LDL-C *mg/dl*	0,99	0,99	1,00	0,03
AP *U/l*	1,64	1,15	2,36	0,01
Biomarker				
NT-pro-BNP *pg/ml*	1,21	1,08	1,35	0,00
PTH *ng/l*	0,86	0,75	0,99	0,04
CRP *mg/l*	1,24	1,10	1,39	0,00
Troponin T *ng/ml*	1,20	1,03	1,40	0,02
ADMA *umol/l*	4,45	1,81	10,98	0,00
25(OH) vitamin D *ng/ml*	0,75	0,55	1,02	0,07
Osteocalcin *ug/l*	0,85	0,71	1,01	0,06

Abbreviations:

ACEI = Angiotensin-Converting-Enzym Inhibitor; AP = alkaline phosphatase.

PTH = parathyroid-hormone; CRP = C-reactive protein.

ADMA = asymmetric dimethylarginin; 25(OH) vitamin D = cholecalciferol.

**Table 3:** Predictors of ESA resistance

**Predictors**									
	**Model 1**			**Model 2**			**Model 3**		
	**Clinical parameters**			**Model 1 plus routine laboratory**			**Model 2 plus specific biomarkers**		
	**OR**	**95% CI**	**p-value**	**OR**	**95% CI**	**p-value**	**OR**	**95% CI**	**p-value**
Sex (male)	1,72	1.27-2.32	<0.001	1,73	1.22-2.45	0.002	1,54	1.08-2.21	0.017
BMI (kg/m^2^)	0.97	0.94-1.00	0.048	0.96	0.93-0.996	0.03	0,96	0.92-0.99	0.02
dialysis vintage (mo)	0.97	0.95-0.99	0.011						
CHF (%)	1,41	1.04-1.91	0.028	1,34	0.96-1.87	0.08	1,30	0.92-1.84	0.14
ACE-inhibitors (%)	1,32	0.98-1.78	0.071	1,33	0,96-1,84	0.09			
heart rate (bpm)	1,01	1,00-1,02	0.052	1,01	1.00-1.02	0.029			
albumin (g/dl)				0,29	0.17-0.52	<0.001	0,40	0.22-0.72	0.002
LDL-cholesterol (mg/dl)				0.996	0.99-1.001	0.125	0.995	0.989-1.001	0.098
creatinine (mg/dl)				1,08	0.99-1.17	0.087			
urea (mg/dl)				0.996	0.991-1.001	0.10			
iron saturation (%)				0,94	0.93-0.96	<0.001	0.95	0.93-0.97	<0.001
potassium (mmol/l)				1,21	0.99-1.47	0.062	1,34	1.09-1.64	0.005
CRP (mg/l)							1,18	1.02-1.38	0.028
Troponin T (ng/ml)							1,17	0.97-1.42	0.10
ADMA (umol/l)							5,24	1.75-15.70	0.003
Osteocalcin (*ug/l*)							0,82	0.66-1.01	0.064

### Predictors of ESA resistance from routine laboratory assessments

#### Multivariate analysis for clinical parameters + routine laboratory

In the next step, we investigated routinely used laboratory variables. The univariate analyses revealed that lower iron saturation, ferritin, albumin, LDL cholesterol and urea concentrations, as well as higher potassium, glucose and alkaline phosphatase concentrations were associated with ESA resistance (Table 2). Again, these parameters were selected for inclusion in the multivariate model. After the application of backward selection procedures, albumin, urea, iron saturation, potassium and creatinine remained the strongest laboratory parameters to predict ESA resistance (Table 3). Concentrations of calcium, phosphate, HbA1c, total and HDL-cholesterol, triglycerides, and platelet count did not associate with ESA resistance in the present analyses.

### Predictors of ESA resistance from specific biomarker assessments

#### Multivariate analysis for clinical parameters + routine laboratory + specific biomarkers

Biomarkers that were significantly associated with ESA resistance in univariate analyses included NT-pro-BNP, PTH, high sensitive CRP, troponin T, ADMA, 25(OH) vitamin D and osteocalcin (Table 2). When these were incorporated into the multivariate analyses, only ADMA, CRP and osteocalcin remained and slightly further improved the risk stratification beyond clinical parameters and routine laboratory (Table 3);

## Discussion

In this post-hoc analysis from the 4D study we developed a prediction model for ESA resistance with easily obtainable clinical parameters, routinely collected laboratory variables and non-routinely used biomarkers. Multivariate analysis for clinical parameters revealed male sex, lower BMI and dialysis vintage, a higher heart rate, the presence of CHF and the use of ACEI as independent predictors for ESA resistance. Among routine laboratory markers, lower concentrations of albumin, iron saturation and higher concentrations of potassium and creatinine were independently associated with ESA resistance and significantly improved risk stratification, when added to the information derived from clinical parameters. Specific biomarkers however did not further improve the risk stratification for ESA resistance.

In the present study, older age and male sex were univariate predictors of ESA resistance. Our results confirm previous findings by Panichi and colleagues [7] which showed that patients who belonged to the highest ERI group were older and predominantly male [7]. Similarly, in an observational study of 1710 patients, ESA resistance was associated with older age, but in contrast to our findings, female sex was a risk factor for ESA resistance in this study [8]. Age and sex however are unmodifiable risk factors. Thus, the identification of potentially modifiable factors is of particular interest.

Several clinical studies have demonstrated that lower albumin levels, decreased BMI as well as increased CRP level are associated with ESA resistance in HD patients [7,8,15,16]. The joint occurrence of malnutrition and inflammation in HD patients is consistent with protein energy wasting [17]. Moreover, nearly one-third of all HD patients have mild to moderate wasting [17] and wasting is associated with sudden cardiac death in these patients [18].

In the current study, lower albumin level and a decreased BMI as well as higher CRP levels independently predicted ESA resistance. As a consequence, strategies to improve nutritional status and to lower the burden of inflammation are likely to play an important role in order to avoid ESA resistance as well as to prevent cardiovascular events in these patients.

It is widely accepted that iron deficiency in HD patients is a strong risk factor for the development of ESA-resistance [4,19], which is consistent with our findings. The investigators of the “European Survey on Anemia Management” (ESAM) study found inadequate iron stores in more than fifty percent of all patients treated with ESAs [20]. Moreover, in a study by DeVita and colleagues, lower doses of ESAs were required to reach target Hb levels, if patients had higher-than-average ferritin levels [21].

In 4D, a further independent risk factor for ESA resistance was treatment with an ACEI. This is consistent with findings in 1513 patients from a post hoc analysis of the RENAAL study [22]. Mohanram and colleagues showed that losartan (ARB) treatment was associated with a significant decrease in hemoglobin level [22]. ACEI and ARBs reduce levels of the peptide hormone angiotensin II. Angiotensin II, besides its more widely known effects on the cardiovascular system, also acts as a growth-factor for the maturation of erythrocytes within the bone marrow. Thus, inhibition of this growth-factor by ACEI and ARBs negatively affects erythropoiesis. On the other hand, in a cross-sectional study in 515 patients by Saudan and colleagues the authors could not demonstrate that the use of ACEI was associated with ESA resistance [23]. However, most clinicians would argue that the positive effects of ACEI or ARBs on the cardiovascular system preponderate any negative effects on erythropoiesis.

In our study, higher potassium levels were associated with resistance to ESAs. Patients in whom it is difficult to maintain potassium levels within the physiological range are often inadequately dialysed. This group of patients are frequently found to suffer from malnutrition (e.g. low BMI) and an increased inflammatory state, which are also, associated with ESA resistance. Furthermore, in 4D we found that the use of ACEI is associated with higher potassium levels. Therefore, the link between higher potassium levels and ESA resistance might be confounded by a higher use of ACEI’s.

The best accuracy in predicting ESA resistance was reached by adding specific non-routinely used biomarkers to the analysis. However, the improvement in risk discrimination was small. These biomarkers are expensive and not routinely measured. In this context, we want to point out that ESA resistance in diabetic HD patients could be well predicted with similar accuracy by including easily obtainable clinical parameters and routinely assessed laboratory variables. We clearly demonstrated that patients could be classified according to risk of ESA-resistance with routinely used parameters.

Several limitations are apparent in the present study. It was a post-hoc analysis within a selected cohort of dialysis patients with type 2 diabetes mellitus. Therefore, the results may not be generalizable to other patient populations, and future studies need to validate our findings in separate cohorts. Second, further parameters that may be of additional interest in improving the risk prediction of ESA resistance such as parameters of oxidative stress and hepcidin were not available. Third, calculating ERI from single ESA and hemoglobin value does not account for the dynamics of the erythropoiesis process. The strengths of this study include the large sample size and little amount of missing values only in a comprehensive and standardized data assessment.

## Conclusions

We provide a prognostic model for ESA resistance based on easily obtainable clinical parameters and routine laboratory markers, which allows accurate identification of diabetic HD patients at risk of ESA resistance. Specific biomarkers did not meaningfully further improve the risk prediction of ESA resistance. We suggest that routinely obtained data can be used in clinical practice to stratify patients according to the risk of ESA resistance, which may help to assign appropriate treatment strategies.

## Competing interests

The authors declare that they have no competing interests.

## Authors’ contributions

AS and CD contributed to every aspect of this article. MPS, AGJ, and CW contributed to the study design, discussion, research data and editing of the manuscript. All authors read and approved the final manuscript.

## Pre-publication history

The pre-publication history for this paper can be accessed here:

http://www.biomedcentral.com/1471-2369/14/67/prepub
